# Initial experience with orbital atherectomy in a tertiary centre in the Netherlands

**DOI:** 10.1007/s12471-022-01742-3

**Published:** 2022-12-12

**Authors:** Wijnand K. den Dekker, Anastasios-Alexandros Siskos, Jeroen M. Wilschut, Rutger-Jan Nuis, Paola Scarparo, Tara Neleman, Kaneshka Masdjedi, Jurgen M. R. Ligthart, Roberto Diletti, Joost Daemen, Nicolas M. Van Mieghem

**Affiliations:** Department of Cardiology, Thoraxcenter, Erasmus University Medical Center, Rotterdam, The Netherlands

**Keywords:** Orbital atherectomy, Calcification, Coronary artery disease, Intravascular imaging, Optical coherence tomography, Intravascular ultrasound

## Abstract

**Background:**

In January 2021, the Diamondback 360 orbital atherectomy (OA) system received CE mark approval and became available in Europe. The first procedure in Europe was performed at the Thoraxcenter, Erasmus Medical Center, Rotterdam, the Netherlands.

**Aims:**

To report the procedural safety and efficacy of the initial experience with OA in a tertiary care institution in the Netherlands.

**Methods:**

Patients with de novo severely calcified coronary artery disease who were treated with intended invasive imaging-guided OA were included in a prospective single-centre registry. Device success, defined as less than 50% stenosis after OA, and procedural success, defined as successful stent implantation with less than 50% residual stenosis, were evaluated. Calcium debulking effects were assessed by invasive imaging. Safety was assessed up to 30 days after the index procedure.

**Results:**

Between February 2021 and June 2021, 29 patients with a total of 39 coronary arteries underwent OA. Target lesions were heavily calcified with a mean length of 32 mm and a calcium arc of 320 degrees. Invasive imaging was applied in all but one patient and 36 vessels. Superficial sanding was observed in almost all vessels (90%) and fracturing of deeper medial calcium in more than half of the vessels (63%), with a device success of 66% and procedural success of 94%. The mean stent symmetry index was 0.84, indicating good circular stent expansion. No primary safety events occurred during 30 days of follow-up.

**Conclusion:**

Our initial experience with OA for heavily calcified coronary lesions demonstrated favourable debulking effects and plaque modification, with high procedural success and clinical safety.

**Supplementary Information:**

The online version of this article (10.1007/s12471-022-01742-3) contains supplementary material, which is available to authorized users.

## What’s new?

- The Diamondback 360 orbital atherectomy (OA) system uses a 1.25-mm, diamond-coated crown that spins around its axis to modify calcified lesions by superficial sanding and fracturing of deeper medial calcium.
- The system received CE mark approval in 2021, and the first procedure in Europe was performed at the Erasmus Medical Center, Rotterdam.
- We report initial experience with this novel device, showing high rates of superficial sanding (90%), fracturing of deeper medial calcium (63%) and ablation of calcified nodules (94%).
- There was no occurrence of coronary perforation or the no-reflow phenomenon, in line with the low rates reported in the ORBIT I and II trials.

## Introduction

Percutaneous coronary intervention (PCI) is increasingly attempted in patients who are of advanced age or have comorbidities that may coincide with excessive coronary artery calcification. Calcified coronary lesions are associated with worse overall outcome [1, 2]. Therefore, calcium modification is paramount for proper stent deployment to avoid underexpansion and/or malapposition and to mitigate the risk of accelerated neoatherosclerosis, stent thrombosis and the need for reintervention [3–6]. Different concepts exist for the modification of coronary calcifications, but formal recommendations are lacking [7]. Orbital atherectomy (OA) with the Diamondback 360 system (Cardiovascular Systems, Inc., St Paul, MN, USA) features a relatively new approach to calcium modification that was originally introduced to treat calcium in peripheral arterial lesions [8].

The orbital atherectomy device (OAD) received U.S. Food and Drug Administration approval in 2013 following the first-in-human ORBIT I [9] and pivotal ORBIT II [10] trials, which showed the safety and efficacy of treating de novo heavily calcified coronary lesions. Approvals in Asia and the Middle East followed in 2018 and 2019, respectively, and a CE mark was granted in January 2021. On 2 February 2021, the first patient with calcified coronary artery disease to be treated with an OAD in Europe underwent the procedure at the Erasmus University Medical Center. Herein, we report our initial series of consecutive patients with calcified coronary artery disease who underwent intended invasive imaging-guided, OA-facilitated PCI.

## Methods

### Patients

This was a prospective single-centre registry to evaluate the safety and efficacy of an OAD in a tertiary care center in the Netherlands. All patients with severely calcified coronary arteries who underwent intended invasive imaging-guided OA between February 2021 and June 2021 were included. Characterisation of severely calcified lesions was based on angiography, defined as the presence of radiopacities noted without cardiac motion prior to contrast injection involving both sides of the arterial wall. The presence of severe calcification was verified by imaging, without any specific inclusion criteria.

### Study device and procedure

**Fig. 1:**
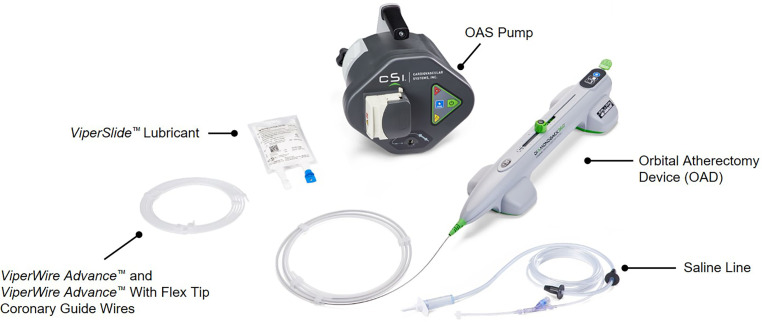
The components of the Diamondback 360 orbital atherectomy system (©2022 Cardiovascular Systems, Inc., St Paul, MN, USA). The image is reproduced with the permission of Cardiovascular Systems, Inc. CSI, Diamondback 360, ViperWire Advance and ViperSlide are trademarks of Cardiovascular Systems, Inc

The OA system consists of four components (Fig. 1): (1) a dedicated 0.012″ guidewire with a nitinol coating and stainless steel core and a 0.014″ tip (ViperWire Advance; Cardiovascular Systems), (2) the OAD, which incorporates a 1.25-mm diamond-coated crown that spins around its axis at 80,000 or 120,000 rpm, (3) a power-providing pump and (4) a dedicated lubricant (ViperSlide; Cardiovascular Systems) that is infused into the system to reduce friction between the OAD and the ViperWire. Balloon dilatation was not allowed prior to OA. The OAD was activated at the proximal edge of the lesion and advanced back and forth at 1 mm/s. The target activated clotting time was > 250 s, and atropine administration or temporary pacemaker insertion was at the discretion of the operator.

Antithrombotic strategies followed the most recent European Society of Cardiology (ESC) guidelines on myocardial revascularisation and the focused update on dual antiplatelet therapy in coronary artery disease [11, 12].

### Device and procedural success

Device success was defined as proper device functioning and < 50% residual stenosis of the target lesion after OA (prior to additional balloon or stent therapy) by invasive imaging [intravascular ultrasound (IVUS) or optical coherence tomography (OCT)]. Procedural success was defined as < 50% residual stenosis after successful stent placement as assessed by invasive imaging (IVUS or OCT). Both definitions were adapted from the first-in-human ORBIT I and II trials [9, 10].

### Invasive imaging

Intravascular imaging with OCT or IVUS was strongly recommended before and after OA and after stent deployment to appreciate the distribution, extent, length and arc of calcium and to evaluate OA (superficial sanding, intimal and deeper medial calcium fracturing and removal of calcified nodules) and stenting results. Optimal stent expansion was assessed according to the MUSIC criteria modified for IVUS and OCT [13].

### Safety

The clinical safety endpoint, major adverse cardiac event (MACE), was defined as a composite of cardiac death, myocardial infarction and target vessel revascularisation at 30 days of follow-up. Cardiac injury was defined according to the new consensus document of the ESC Working Group on Cellular Biology of the Heart and the European Association of Percutaneous Cardiovascular Interventions.

## Results

### Patient demographics

Between 2 February and 12 May 2021, 29 patients with 39 severely calcified vessels were treated with OAD and included in the registry. Baseline characteristics are shown in Table S1 (Electronic Supplementary Material). Mean age was 71.5 ± years and 55% were male. Traditional risk factors for coronary artery disease and calcified lesions were present. The clinical context was chronic coronary syndrome in 19 patients (65.6%), unstable angina in 3 patients (10.3%) and non-ST-elevation myocardial infarction in 7 patients (24.1%).

### Procedural characteristics

Procedural characteristics are shown in Tab. 1. Radial access was used in all patients and sheath size was 6 Fr in all but two (6.5 Fr sheathless system in one patient and 7 Fr in another). Haemodynamic support was used upfront in one patient and as a bailout procedure in two patients because of haemodynamic instability during the procedure. In all three patients, mechanical circulatory support was discontinued within 24 h. Mean procedure time was 126 min and a mean of 152 cc of contrast was used.

**Table 1:** Procedural characteristics

Variable	Total (*n* = 29)
*Access site*	
– Radial	29/29 (100.0)
– Femoral	0/29 (0.0)
Catheter size (Fr)	6 ± 0.4
*Imaging mode*	28/29 (96.6)
– IVUS	13/29 (44.8)
– OCT	17/29 (58.6)
Total number of arteries treated per patient	1.7 ± 0.7
Number of arteries treated with OAS per patient	1.4 ± 0.6
*Haemodynamic support*	3/29 (10.3)
– Inotropes	1/29 (3.4)
– IABP	2/29 (6.9)
– pLVAD	1/29 (3.4)
*Timing of haemodynamic support*	
– Upfront	1 (33.3)
– Bailout	2 (66.7)
Stent length (mm)	105.5 ± 51.0
Number of stents implanted	3.8 ± 1.8
Procedure time (min)	126.7 ± 39.0
Contrast (ml)	152.6 ± 60.1
Total area dose (cGy cm^2^)	5564.6 ± 3489.3
Total skin dose (mGy)	1191.8 ± 736.5

Values are mean ± standard deviation or *n* (%)

*IVUS* intravascular ultrasound, *OCT* optical coherence tomography, *OAS* orbital atherectomy system, *IABP* intra-aortic balloon pump, *pLVAD* percutaneous left ventricular assist device

**Fig. 2:**
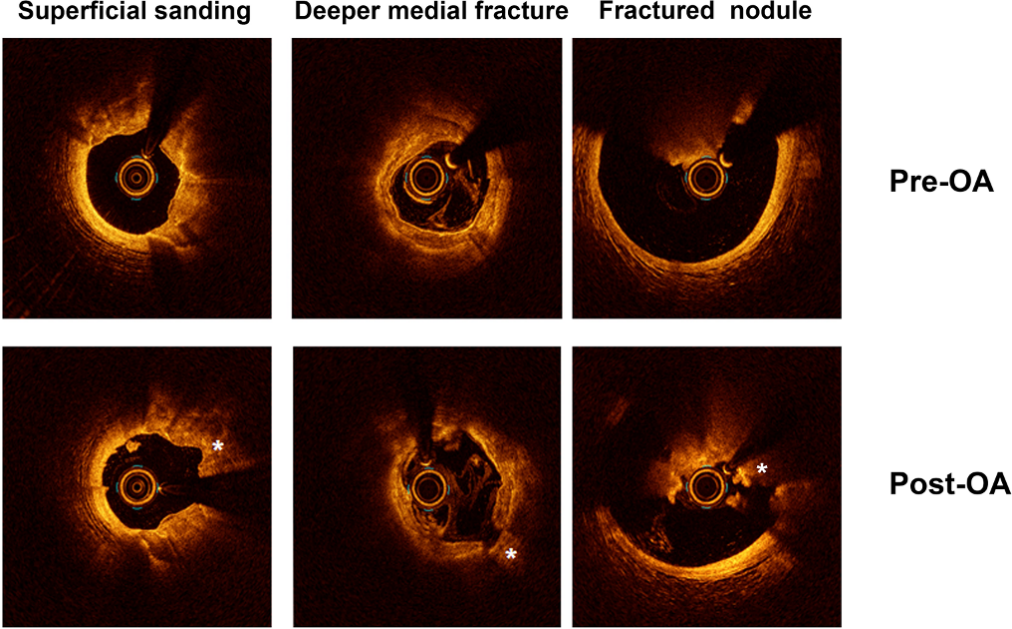
Example of superficial sanding, medial calcium fracture and fractured calcified nodule. *OCT* optical coherence tomography. *Asterisks* indicate the different modification patterns of orbital atherectomy (*OA*)

Overall, 39 coronary arteries were treated with OA, including 18 lesions in the left anterior descending artery, 15 lesions in the right coronary artery and 6 lesions in the left circumflex artery (Tab. 2). Thirty-six lesions were deemed type C lesions, one lesion was type B2 and two lesions were type B1. Recommended invasive imaging was performed in all but one patient. OCT was used in 20 vessels, IVUS in 16 vessels, a combination of OCT and IVUS in 2 vessels and in 3 vessels no imaging was performed. Complete matching of the treated vessel, pre-OA, post-OA and post-stenting was available for 26 vessels. A final invasive imaging study to determine procedural success was available in 29 lesions. Mean arc and length of calcium were 278.0 ± 104.8 degrees and 35.1 ± 19.6 mm, respectively, while mean calcium thickness was 1.03 ± 0.26 mm. Minimal luminal diameter was 1.6 ± 0.3 mm, while the reference vessel diameter was 3.1 ± 1.1 mm. All but one lesion was successfully treated at the lower speed setting of 80,000 rpm. One severely calcified right coronary artery was treated at both 80,000 and 120,000 rpm (Tab. 3). Device success was achieved in 19 of 29 lesions (66%) with superficial sanding of calcium in 27 of 30 lesions (90%) and fracturing of deeper medial calcium in 19 of 30 lesions (63%). Fig. 2 shows examples of superficial sanding, medial calcium fracture and ablation of calcified nodules. Noteworthy is that OA appeared to be particularly effective in the ablation of calcified nodules, with 94% of nodules being removed. After OA, additional balloon dilatation preceded stent placement in all vessels. A mean of 2.3 ± 1.0 stents were implanted per vessel with a mean overall stented length of 64.3 ± 27.0 mm. Postdilatation was performed in 35 vessels. All treated vessels had TIMI (thrombolysis in myocardial infarction) grade III flow at the end of the procedure. There were no coronary perforations. Procedural success was 96% as assessed by invasive imaging. Mean minimal luminal area post-stenting was 4.9 ± 1.5 mm^2^, with an average stent symmetry index per treated vessel of 0.84 ± 0.03.

**Table 2:** Characteristics of vessels treated with orbital atherectomy

Variable	Total (*n* = 39)
*Target vessel*	
– LAD	18/39 (46.2)
– RCA	15/39 (38.5)
– LCx	6/39 (15.4)
*Lesion classification*	28/29 (96.6)
– A	0/39 (0.0)
– B1	2/39 (5.1)
– B2	1/39 (2.6)
– C	36/39 (92.3)
*Imaging mode*	36/39 (92.3)
– IVUS	20/39 (3.4)
– OCT	18/39 (6.9)
Calcium visible on angiography	36/39 (92.3)
Calcium arc (degrees)	278.0 ± 104.8
Calcium length (mm)	35.1 ± 19.6
Calcium thickness (mm)	1.03 ± 0.26
Superficial calcium	30/39 (100)
Deeper medial calcium	17/39 (56.7)
Minimal luminal diameter (mm)	1.6 ± 0.3
Reference vessel diameter (mm)	3.1 ± 1.1

Values are mean ± standard deviation or *n* (%)

*LAD* left anterior descending artery, *RCA* right coronary artery, *LCx* left circumflex artery, *IVUS* intravascular ultrasound, *OCT* optical coherence tomography

**Table 3:** Procedural characteristics of vessels treated with orbital atherectomy (*OA*)

Variable	Total (*n* = 39)
*OA device speeds(rpm)*	
– 80,000	38/39 (100)
– 120,000	0/39 (0.0)
– 80,000 and 120,000	1/39 (2.6)
Number of runs	7 (3–26)^a^
Run time (s)	171.8 ± 108.4
Superficial sanding	27/30 (90)
Medial fractures	19/30 (63)
Removal of calcified nodules	15/16 (94)
Device success	19/29 (66)
Predilatation	39/39 (100)
Postdilatation	35/39 (89.7)
Stent implanted	39/39 (100)
Stent length (mm)	64.3 ± 27.0
Number of stents implanted	2.3 ± 1.0
Procedural success	27/28 (96)
Minimal stent area (mm^2^)	4.9 ± 1.5
Stent symmetry	0.84 ± 0.04
*Post-procedure TIMI flow*	
– I	0/39 (0.0)
– II	0/39 (0.0)
– III	39/39 (100)

Values are mean ± standard deviation or *n* (%)

*TIMI* thrombolysis in myocardial infraction

^a^Mean (minimum and maximum)

### Safety

No clinical safety events occurred during 30 days of follow-up (Table S2, Electronic Supplementary Material). Cardiac enzymes were collected in 19 of 22 patients who were treated for (un)stable angina. Minor myocardial injury [high-sensitivity troponin T (hsTNT) > 1 × 99th percentile of upper reference limit (URL) ≤ 5 × 99th percentile URL] was noted in 5 of 19 patients (26.3%) and major myocardial injury (hsTNT > 5 × 99th percentile URL) in 13 of 19 patients (68.4%). No patients suffered from periprocedural (type 4a) myocardial infarction [14].

## Discussion

This initial single-center experience with OA to modify calcified coronary lesions confirmed appropriate device and procedural success with a favourable clinical outcome at 30 days of follow-up. OA refines coronary calcium through sanding and fracturing of both superficial and deeper medial calcium. The creation of microparticles < 2 µm and the absence of vessel obstruction during therapy may reduce the risk of a no-reflow phenomenon.

Despite a lack of experience in handling the device, coronary lesions in this series appeared more complex than those reported in the pivotal ORBIT I and II registries. Of all lesions, 95% were type B2/C, while the proportion was 44% and 83% in ORBIT I and II, respectively. All calcified lesions were assessed by invasive intracoronary imaging, revealing a mean arc of 278 degrees, with a maximum of 360 degrees and a mean length of 35 mm, values that not only correlate with severe calcification [7, 15] but are also predictors for stent underexpansion [16]. In comparison, in the ORBIT II trial the maximum arc was 295 degrees with a mean calcium length of 29 mm.

A wide armamentarium exists to modify calcified coronary lesions, from plain old balloons to more sophisticated equipment like lasers, lithotripsy, rotational atherectomy (RA) and OA. The goal of these devices is to modify and/or fracture the calcium in order to adequately place a stent. While RA has been available since the late 1980s, OA was not launched in the USA until 2013 and in Europe not until 2021. There are some marked differences between RA and OA. Superficial sanding has become the main mechanism of action of RA, as healthy tissue is able to deflect away from the burr while the calcified plaque cannot do so, resulting in ablation of calcium. This results in smoothening of the luminal surface, without the need for luminal gain. The concept of plaque modification to facilitate balloon angioplasty and stent expansion instead of plaque debulking followed the STRATAS and CARAT trials, which showed more angiographic and clinical complications and more target lesion revascularisations with the use of larger burr sizes (burr/artery ratio > 0.7) [17, 18]. Because the OAD orbits through the lumen, there is less guidewire bias and potentially more luminal gain as compared to RA. Apart from superficial sanding OAD may fracture more deeply located calcium in the tunica media. Indeed, we observed a sanding effect in 27 of 30 vessels and medial calcium fractures in 19 of 30 vessels. Intravascular lithotripsy (IVL) is another calcium modification technique that can fracture deeper medial calcification, through generation of sonic pressure waves. The safety and efficacy of this technique were proven in the DISRUPT CAD I, II and III trials [19–21]. Both RA and OA can be combined with IVL for the treatment of calcified lesions, and both combinations have been described in the literature with good results. From a mechanistic point of view, there seem to be more reasons for combining RA with IVL than for OA with IVL. With RA, deeper medial calcium is not modified but can be treated in addition with IVL. However, due to the orbital nature of the OAD, deeper medial calcium is also modified, which would decrease the need for IVL. In our registry, there was no need for additional treatment with IVL. A particular effect of OA was also seen on calcified nodules that were all abolished, which might be due to less guidewire bias compared to RA. The profound calcium modification resulted in 96% procedural success and < 50% residual stenosis post-stenting in all but one lesion. Coronary perforation and the no-reflow phenomenon did not occur in our series. Our findings echo those of the ORBIT I and II studies, which reported low rates of coronary perforation (2% and 0.9%, respectively) and absence of the no-reflow phenomenon. In a recent retrospective study [22], coronary perforation was reported to be higher with OA than with RA, which might be due to the orbital nature of the OAD. The particles that arise from superficial sanding are small (< 2 μm) and, combined with the continuous antegrade flow, this leads to less distal embolisation and microvascular obstruction. As a result, there is a lower risk of no-reflow compared to RA, which is characterised by larger microparticles (5–10 μm) and burr-induced flow obstruction during therapy delivery.

In the ORBIT I and II trials, the rate of MACE at 30 days was 6% and 10.4%, respectively. In comparison, we found no major clinical events in our series, although minor cardiac damage was seen in 5 of 19 patients and major cardiac damage was seen in 13 of 19 patients in whom serial biomarkers were available. The randomised ECLIPSE trial (NCT03108456) compares the clinical outcome in patients with severely calcified coronary artery disease treated with an OA-based strategy with those undergoing conventional angioplasty. A total of 2000 patients will be randomised in 114 centres in the United States. The primary endpoint is target vessel failure, defined as a composite of cardiac death, target vessel-related myocardial infarction or ischaemia-driven target vessel revascularisation. A European prospective trial will explore in depth the effect of OA on severely calcified coronary lesions. One hundred patients in six centres in the Netherlands, Germany and Italy will undergo OCT-guided treatment. The primary endpoint will be stent expansion as assessed by OCT-derived minimal stent area, while secondary endpoints will include the number of calcium fractures, final minimal stent area, minimal lumen area post-OA and post-stenting, incidence of dissections and haematomas post-OA.

### Limitations

The findings are based on early experience with OA at a single centre in the Netherlands with possible selection bias. Nevertheless, the coronary lesions were complex and heavily calcified. Moreover, the high device and procedural success in addition to the low complication rate underscored the safe and steep learning curve during the adoption of the OAD technology in our practice. Imaging data were site reported but reported by experienced imagers who were blinded as regards the procedure and the clinical outcome of the patients.

## Conclusion

This initial experience with OA for heavily calcified coronary lesions demonstrated favourable debulking effects and plaque modification, with high procedural success and clinical safety.

## Supplementary Information

Table S1 Baseline characteristics

Table S2 Major Adverse Cardiac Events (MACE) at 30 days
