# Supplementary material for: Initial experience with orbital atherectomy in a tertiary centre in the Netherlands
Source: Neth Heart J. 2022 Dec 12;31(5):196–201. doi: 10.1007/s12471-022-01742-3 (PMC10140256; doi:10.1007/s12471-022-01742-3)
Supplement: Supplementary file 1 — Table S1 Baseline characteristics [file 12471_2022_1742_MOESM1_ESM.docx]

**Table S1 Baseline characteristics**

| Variable | Total  (n=29) |
| --- | --- |
| Age (years) | 71.5 ± 7.7 |
| Male | 16/29 (55.2) |
| Hypertension | 24/29 (82.8) |
| Hypercholesterolemia | 20/29 (69.0) |
| Diabetes | 8/29 (27.6) |
| Family History | 7/29 (24.1) |
| Current Smoker | 7/29 (24.1) |
| Previous Stroke | 3/29 (10.3) |
| Previous MI | 8/29 (27.6) |
| Previous PCI | 9/29 (31.0) |
| Peripheral Artery Disease | 11/29 (37.9) |
| Chronic Kidney Disease | 3/29 (10.3) |
| CABG | 1/29 (3.4) |
| Clinical Presentation |  |
| Stable Angina | 19/29 (65.5) |
| Unstable Angina | 3/29 (10.3) |
| NSTEMI | 7/29 (24.1) |
|  |  |

Values are mean ± standard deviation or n (%).
MI = Myocardial Infarction; PCI = Percutaneous Coronary Intervention; CABG = Coronary Artery Bypass Grafting; NSTEMI = Non-ST-Elevation Myocardial Infarction; PCI = percutaneous coronary intervention.
