# Supplementary material for: Initial experience with orbital atherectomy in a tertiary centre in the Netherlands
Source: Neth Heart J. 2022 Dec 12;31(5):196–201. doi: 10.1007/s12471-022-01742-3 (PMC10140256; doi:10.1007/s12471-022-01742-3)
Supplement: Supplementary file 2 — Table S2 Major Adverse Cardiac Events (MACE) at 30 days [file 12471_2022_1742_MOESM2_ESM.docx]

**Table S2 Major Adverse Cardiac Events (MACE) at 30 days**

Values are n (%).

TVR = Target Vessel Failure.

| Outcome | In-hospital | 30-Day Follow Up |
| --- | --- | --- |
| MACE | 0/29 (0.0) | 0/29 (0.0) |
| Cardiac Death | 0/29 (0.0) | 0/29 (0.0) |
| Myocardial Infarction | 0/29 (0.0) | 0/29 (0.0) |
| TVR | 0/29 (0.0) | 0/29 (0.0) |
| Minor Myocardial Injury | 5/19 (26.3) |  |
| Major Myocardial Injury | 13/19 (68.4) |  |
